# Identifying the important factors associated with teaching sex education to people with intellectual disability: A cross-sectional survey among paid care staff^†^

**DOI:** 10.3109/13668250.2014.899566

**Published:** 2014-03-28

**Authors:** Dilana Schaafsma, Gerjo Kok, Joke M. T. Stoffelen, Paulien Van Doorn, Leopold M. G. Curfs

**Affiliations:** ^1^ Department of Work and Social Psychology, Faculty of Psychology and Neuroscience, Maastricht University, Maastricht, the Netherlands; ^2^ Governor Kremers Centre, Maastricht, the Netherlands; ^3^ Lunet zorg, Eindhoven, the Netherlands; ^4^ Department of Clinical Genetics, Faculty of Health, Medicine and Life Sciences, Maastricht University, Maastricht, the Netherlands

**Keywords:** sex education, intellectual disability, paid care staff, needs assessment

## Abstract

**Background:**

Sex education programs have been developed with paid care staff as sex educators. However, no information is available about whether these programs are being delivered.

**Method:**

The aim of this study was to investigate whether paid care staff working in an organisation specialised in the care of people with mild to moderate intellectual disability teach sex education or not. An online questionnaire was therefore constructed to assess the important factors associated with teaching sex education.

**Results:**

Of the 163 staff members who completed the questionnaire, 39% provided sex education. Results show that it was mainly provided reactively. The main factor was the perceived social norm towards teaching sex education.

**Conclusions:**

If we want paid care staff to teach sex education reactively, then we need to focus on changing the perceived social norm. However, if we want them to teach sex education proactively, a new needs assessment should be conducted in order to identify the important factors to motivate and enable them to provide sex education.

## Introduction

Intellectual disability is characterised by significant limitations both in intellectual functioning and in adaptive behaviour as expressed in conceptual, social, and practical adaptive skills (Luckasson & Schalock, 2013; Schalock et al., 2010, 2012; Schalock & Luckasson, 2013). Regardless of this impairment in intellectual functioning, people with intellectual disability have sexual needs and desires, like anyone else. In several studies, individuals with intellectual disability have expressed their desire to have intimate relationships, to marry, and to have children (Healy, McGuire, Evans, & Carley, 2009; Kelly, Crowley, & Hamilton, 2009). However, due to their disability, they encounter more barriers in the area of sexuality and are more vulnerable to sexual abuse than people without disability (Eastgate, van Driel, Lennox, & Scheermeyer, 2011; McCarthy, 1996; Stoffelen, Kok, Hospers, & Curfs, 2013; Yacoub & Hall, 2009). People with intellectual disability have been found to have insufficient or incorrect knowledge about subjects like masturbation, pregnancy, safe sex, reproduction, and same-sex relationships (Healy et al., 2009; Kelly et al., 2009; Leutar & Mihoković, 2007; McCarthy, 2009; Murphy & O'Callaghan, 2004). Furthermore, they experience a lack of privacy (e.g., people are more likely to enter their room without knocking), receive restrictive rules concerning intimate relationships, or experience people expressing disapproval towards them having an intimate relationship (Healy et al., 2009; Kelly et al., 2009). These problems affect the sexual health of people with intellectual disability and, consequently, their quality of life.

Sex education programs have been developed in order to improve the sexual health of people with intellectual disability. Sexual health, as defined by the World Health Organization (WHO, 2006), is not merely the absence of disease or negative experiences regarding sexuality, but includes positive aspects as well, such as “the possibility of having pleasurable and safe sexual experiences” (p. 5). Furthermore, WHO's definition states that “the sexual rights of all persons must be respected, protected and fulfilled” (p. 5). In short, all people have the right to experience sexuality in a positive and pleasurable way. Sex education programs in the Netherlands are, in most cases, designed to be delivered by paid care staff (Schaafsma, Stoffelen, Kok, & Curfs, 2013). However, there is no evidence supporting the idea that paid care staff are willing and feel able to successfully deliver sex education to people with intellectual disability. In order to find out whether paid care staff members are willing and feel skilful enough to provide sex education, it is important to know what kind of factors are associated with the provision of sex education by paid care staff.

### Theory of planned behaviour/reasoned action approach

The theory of planned behaviour and its more recent version, the reasoned action approach (Fishbein & Ajzen, 2010), are social cognitive theories that describe the influence of changeable cognitive determinants on human behaviour and can help us explain why paid care staff members do or do not teach sex education. Determinants are those factors that have been found to be associated with the performance of the behaviour of the target population, and it is assumed that there is a causal relationship between the determinants and the behaviour (Bartholomew, Parcel, Kok, Gottlieb, & Fernández, 2011). Among these determinants, the intention to perform the desired behaviour is the most important determinant of future behaviour. However, the intention to perform the desired behaviour is, in turn, determined by the attitude towards the behaviour, the perceived social norms regarding the behaviour, and the perceived self-efficacy (similar to perceived behavioural control and perceived competence) in terms of performing the desired behaviour.

Attitude refers to the person's overall evaluation of the desired behaviour change and includes both the positive and negative consequences of the behaviour. Two types of attitude have been identified in the literature. The instrumental attitude is based on an evaluation of the advantageous or disadvantageous outcomes of a certain behaviour. Experiential attitude, on the other hand, is an affective evaluation of the outcomes of a certain behaviour (e.g., this behaviour feels comfortable or uncomfortable; Bartholomew et al., 2011, p. 75). Perceived social norms refer to the person's beliefs about whether important people, for example, colleagues, approve of the desired behaviour or not. Two types of social norms are distinguished. An injunctive social norm refers to a person's perception of what behaviour is approved of or disapproved of by a specific group of people within a specific context. A descriptive social norm refers to a person's perception of what behaviour is commonly exerted by a specific group of people within a specific context (Bartholomew et al., 2011, p. 129). Perceived behavioural control, similar to perceived self-efficacy, refers to the person's perceived skills to perform the desired behaviour (Bartholomew et al., 2011, p. 64). These social cognitive models of behaviour thus imply that paid care staff members will be willing to provide sex education if they demonstrate (a) a positive attitude towards providing sex education; (b) perceive social support and approval from important others in the work context, including colleagues, managers, and clients’ parents; and (c) feel confident and capable of providing the intended sex education.

Although the reasoned action approach uses the term *determinant*, the current study only tested for correlations and therefore cannot demonstrate causality and so instead uses the term *factors* or *factors associated with* when referring to personal or environmental determinants.

### Potential barriers

Previous studies show that paid care staff do not always have positive attitudes about sexuality, feel skilful enough to teach sex education, or feel supported by their environment regarding sex education. With regard to attitudes, older staff members seem to have more conservative attitudes towards the sexuality of their clients than younger staff members (Abbott & Howarth, 2007; Evans, McGuire, Healy, & Carley, 2009; Gilmore & Chambers, 2010; Meaney-Tavares & Gavidia-Payne, 2012), but the general attitude of staff is more liberal and in general positive compared to the attitude of family members (Bazzo, Nota, Soresi, Ferrari, & Minnes, 2007; Evans et al., 2009; Gilmore & Chambers, 2010).

Regarding self-efficacy, several studies show that staff members reported a lack of experience in dealing with sexuality (Abbott & Howarth, 2007) and also a lack of training in this area (Abbott & Burns, 2007; Evans et al., 2009; Grieve, McLaren, & Lindsay, 2007).

When it comes to the perceived social norm, some staff members expressed concerns about the reactions of other staff members or parents if they were to talk to clients about sexuality (Abbott & Howarth, 2007).

Furthermore, sex education programs are designed to be delivered proactively (Schaafsma et al., 2013). This means that sex education should be provided before the person with intellectual disability is sexually active, so that the person has the knowledge, cognition, and skills needed to make decisions that have a positive effect on his or her sexual health. When a person lacks the right knowledge, cognition, or skills, problems may arise that have a negative impact on his or her sexual health. For example, the person with intellectual disability may be more vulnerable to sexual abuse, due to a lack of skills needed to identify abusive situations (Murphy & O'Callaghan, 2004). Previous studies, however, show that staff in general teach sex education reactively (Abbott & Burns, 2007; Abbott & Howarth, 2007) and respond idiosyncratically (Evans et al., 2009) to situations concerning sexuality issues, which is a cause for concern.

Next to cognitive determinants, environmental determinants can also play an important role. Environmental determinants can form barriers to performing the desired behaviour. For example, policies on sexuality within organisations are often inadequate or simply not present (Abbott & Howarth, 2007), or staff may be unfamiliar with the policy (Cuskelly & Bryde, 2004) or unsure about how to implement the policy (Grieve et al., 2007). Some staff members even worry about being prosecuted if they attempt to discuss sexuality with their client (Grieve et al., 2007). A lack of policy or ambiguities about the organisation's policy may be a possible barrier to teaching sex education, even when staff members express a positive intention.

### Present study

In the present study, we investigated the differences between paid care staff members who teach sex education to people with intellectual disability and paid care staff members who do not teach sex education. We hypothesised that paid care staff behaviour relating to sexual health education would be directly and indirectly influenced by a range of personal and environmental issues.

The goal of the study was to identify important factors and possible barriers (independent variables) associated with teaching sex education to people with intellectual disability (dependent variable).

## Methods

### Ethical procedures

Approval for the study was given by the Ethical Committee of the Faculty of Psychology and Neuroscience at Maastricht University, the Netherlands. Preceding the online questionnaire, participants were provided with information about the aims of the study. They were also informed that participation was anonymous and that they could discontinue their participation at any time.

### Participants and procedure

For this study we used a convenience sample. The organisation where the study was conducted is a research partner. This organisation is specialised in the care of people with intellectual disability. In the Netherlands, organisations such as this one accommodate a large portion of the population of people with intellectual disability and are therefore important providers of sexual health care. In 2008 the number of people who requested extra support services from these organisations was 147,000 (Ras, Woittiez, van Kempen, & Sadiraj, 2010).

All paid care staff members were sent a link to an online questionnaire (*N* = 1475). A total of 630 paid care staff members completed the questionnaire, resulting in a response rate of 43%. This number was reached 2 weeks after the questionnaire was placed online, with a reminder sent after 1 week. For the analyses, 163 of the 630 paid care staff members were included. This group only included people who were directly responsible for the wellbeing of the client. They provided inpatient care to people with a moderate or mild intellectual disability, did not work exclusively with children under the age of 12, and did not work at an activity centre (part of the organisation that provides activities for clients during the day). According to the guidelines of the organisation, the selected sample was responsible for the sexual health of their clients, and therefore most eligible to provide sex education. This means that the staff members who were excluded from the study worked, for example, with the elderly or with children, had clients who did not live within the facilities of the organisation, or worked with clients who had a severe or profound intellectual disability.

### Measures

Except for age, behaviour, presence of sex education materials, and descriptive social norm, constructs were measured using a 5-point scale item, where 1 = *totally disagree* and 5 = *totally agree.* Items for intention, self-efficacy, attitude, and social norms were constructed using the guidelines provided by Fishbein and Ajzen (2010). In accordance with these guidelines, the content of the questions was derived from the results of qualitative research; in this case, 10 interviews conducted among a representative sample of paid care staff members (Table 1). Pearson correlation is used for scales with two items. Omega total (ω_*t*_) is used to indicate the reliability of scales with more than two items (Revelle & Zinbarg, 2009) instead of the widely used Cronbach's alpha, because Cronbach's alpha has been shown to be, in many cases, a gross underestimate of reliability. Moreover, Cronbach's alpha is used in many papers as a measure for internal consistency, but is unrelated to the internal structure of a test (Sijtsma, 2009). A paper by Revelle and Zinbarg (2009) revealed that ω_*t*_ is a better estimate of reliability than Cronbach's alpha or other estimates such as the greatest lower bound.

**Table 1.** The constructs measured by the survey

Variable/topic	Number of items	Reliability	Example question/statement
Behaviour	1	n/a	Do you teach sex education?
Intention	2	*r* = .96	Do you intend to teach sex education in the next 3 months?
Self-efficacy	1	n/a	I think I will succeed, when I teach a client sex education.
Instrumental attitude	3	*ω_t_* = .95	Teaching people with mild or moderate intellectual disability sex education is: important/not important.
Experiential attitude	2	*r* = .44	Teaching people with mild or moderate intellectual disability sex education is: hard/easy.
Injunctive social norm	4	*ω_t_* = .98	Most of my colleagues think it is important that I teach sex education.
Descriptive social norm	1	n/a	Indicate the percentage of colleagues that teach sex education.
Feelings of professional responsibility	1	n/a	It is my duty, as a professional, to provide sex education to my clients.
Familiarity to the content of the protocols and policy	1	n/a	Indicate to what extent you are familiar with the policy on sexuality within [name of organisation].
Education	2	*r* = .73	I receive sufficient training in the area of sexuality and intellectual disability.
Informational support	5	*ω_t_* = .91	How much informational support do you receive from your colleagues?
Materials	1	n/a	The sex education materials take into account, in most cases, the disability level of the client.
Client behaviour	8	*ω_t_* = .83	Do you have experience with clients having a relationship?
Reasons to teach sex education	10	n/a	The client is developmentally ready.
Reasons not to teach sex education	9	n/a	I don't have time for it.
Belief that sex education can lead to more problems	1	n/a	Teaching sex education to people with intellectual disability leads to more problems in the area of sexuality.

*Note*, n/a = not applicable; *r* = Pearson correlation; *ω_t_* — omega total.

Two health psychologists and a sexologist working in the field of intellectual disability checked the content of the questionnaire. Additionally, five paid care staff members checked the content for feasibility and understandability, and adjustments were made accordingly.

### Analysis

The correlation between intention to teach sex education and past behaviour in terms of teaching sex education was calculated in order to examine the predictive value of intention on past behaviour. Next, bivariate correlations were determined for the study variables and for intention. A multiple regression analysis, using blockwise entry, was conducted to discover the unique contribution of the study variables to the explanation of intention as well as the total amount of variance explained in intention. Psychosocial factors, derived from the reasoned action approach (Fishbein & Ajzen, 2010), were entered first. Second, modifiable factors, derived from the interviews, with a high correlation with intention were added. And finally, client behaviour, which is a measure of experience with matters in the area of sexuality, was added. Furthermore, independent sample *t* tests were carried out to identify differences on individual constructs and single items between staff members who teach sex education and those who do not. Finally, a cross-tabulation was performed to identify a possible relationship between the presence of sex education materials and the behaviour (practice) of teaching sex education.

## Results

### Sample description

The sample (*N* = 163) was 85% female and 15% male, with a mean age of 37.63 (*SD* = 10.87, range: 22–63). Years of working experience ranged from 1 to 42 years, with a mean of 14.48 (*SD* = 9.96). The percentage of people who worked 11–20 hours was 15%; 50% of people worked 21–30 hours, and 35% worked 31–40 hours. Concerning highest education, 1% finished high school, 52% completed intermediate vocational training, 42% higher vocational education, 3% university, and 3% filled in “other.”

### Current behaviour and intention

Of the 163 staff members, 39% (*N* = 64) indicated that they currently teach sex education and 61% (*N* = 99) indicated that they did not teach sex education at the moment of filling out the questionnaire. Intention to teach sex education had a mean of 2.28 (*SD* = 1.46), with a minimum of 1 and a maximum of 5. A strong positive correlation was found between the intention to teach sex education and the past behaviour of teaching sex education, *r*(163) = .75. Scores on intention to teach sex education were higher for staff members that teach sex education (*M* = 3.63, *SD* = 1.27) than for the staff members who do not teach sex education (*M* = 1.41, *SD* = 0.73), *t*(90) = 12.71, *p* < .01, *d* = 2.28.

### Correlations of study variables

Age showed a low positive correlation with both instrumental (.17) and experiential (.21) attitude. Client behaviour (.55), responsibility (.37), injunctive social norms (.58), and descriptive social norms (.49) showed a medium to high positive correlation with intention. Self-efficacy (.17), instrumental attitude (.17), experiential attitude (.21), policy (.28), and education (.22) showed a low to medium positive correlation with intention. All these correlations were significant, with a *p* level of < .05. Age and informational support did not correlate with intention.

Table 2 shows a multiple regression analysis of paid care staff members’ intention to teach sex education in the next 3 months. A model with self-efficacy, instrumental attitude, experiential attitude, injunctive social norms, and descriptive social norms explained 42%, *R*^2^ = .42, of the variance in intention, *F*(5, 157) = 22.97, *p* = < .01. Adding responsibility, policy, education, and informational support increased the explained variance by 2%, *R*^2^ = 44, *F*(9, 153) = 1.30, *p* = .27. Adding client behaviour increased the explained variance to another 7% to *R*^2^ = .51, *F*(10, 152) = 20.21, *p* = .01. In the final regression model, unique significant contributions were found for injunctive social norm, descriptive norm, responsibility, and client behaviour.

**Table 2.** Regression analysis with the intention to teach sex education as outcome variable

		Model 1		Model 2		Model 3	
Factors	*r*	β	*p*	β	*p*	*β*	*p*
RAA variables							
Self-efficacy	.17*	.10	.11	.04	.59	−.01	.88
Instrumental attitude	.17*	.03	.64	.03	.68	.06	.36
Experiential attitude	.21**	.08	.22	.07	.28	.05	.40
Injunctive social norm	.58**	.43	.00	.36	.00	.30	.00
Descriptive social norm	.49**	.28	.00	.28	.00	.19	.01
Additional variables							
Responsibility	.37**			.13	.11	.15	.04
Policy	.28**			.07	.27	−.02	.71
Education	.22**			.02	.72	.02	.74
Informational support	.08			−.05	.42	−.04	.55
Previous experiences							
Client behaviour	.55**					.32	.00
*R*^2^		.42	< .01	.44	< .01	.51	< .01
Δ*R*^2^				.02	.27	.07	< .01

*Note. N* = 163.

* *p* < .05, two-tailed. **p < .01, two-tailed.

### Staff members who teach sex education versus staff members who do not

With regard to the study variables, staff members who indicated that they did teach sex education at the moment of filling out the questionnaire were compared to staff members who did not teach sex education. Staff members who did teach sex education reported higher intentions to teach sex education (*M*_no_ = 1.14, *M_yes_* = 3.63, *p* < .01), rated their environment as being more positive towards teaching sex education (injunctive social norm; *M*_no_ = 2.28, *M*_yes_ = 3.87, *p* < .01), indicated a higher percentage of colleagues that teach sex education (descriptive social norm; *M*_no_ = 14.40, M_yes_ = 40.64, *p* < .01), showed a more positive experiential attitude (*M*_no_ = 3.25, *M*_yes_ = 3.61, *p* <.01), reported higher feelings of responsibility for teaching sex education (*M*_no_ = 3.63, *M*_yes_ = 4.17, *p* < .01), and were more satisfied with the education they received in the area of sexuality (*M*_no_ = 2.76, *M*_yes_ = 3.27, *p* < .01). It was noticeable that high scores for instrumental attitude (*M*_no_ = 4.59, *M*_yes_ = 4.73) and self-efficacy (*M*_no_ = 3.87, *M*_yes_ = 4.08) were found in both groups and did not differ significantly between the two groups.

### Client behaviour

Several sexuality-related matters were presented (Table 3), and paid care staff members were asked to indicate to what extent they had experience with these matters. The data are presented for the group that teaches sex education and the group who does not. Staff members reported having most experience with clients who have a relationship, clients who have problems within their relationship, and clients who exhibit sexually inappropriate behaviour. Staff members who teach sex education reported having more experience on all items than staff members who do not teach sex education, except for the item “client has masturbation-related problems.”

**Table 3.** Differences in experiences between staff members who teach sex education and those who do not on client behaviour items

	Teaching sex education							
	No group (*n* = 99)		Yes group (*n* = 99)					
Experiences	*M*	*SD*	*M*	*SD*	*df*	*T*	*P*	*d*
The client has a relationship	2.56	1.34	3.45	1.15	148	−4.55	< .01	.71
The client has sexuality-related questions	2.14	0.92	2.98	0.85	142	−6.02	< .01	.95
The client has relationship problems	2.20	1.20	3.30	0.99	152	−6.36	< .01	.98
The client wants a child	1.37	0.71	2.13	1.02	102	−5.16	< .01	.89
The client exhibits sexually inappropriate behaviour	2.29	0.96	2.83	0.92	161	−3.53	< .01	.57
The client has been sexually abused	2.22	1.14	2.70	1.12	161	−2.65	< .01	.43
The client has sexually abused a person	1.44	0.88	1.89	0.86	161	−3.19	< .01	.51
The client has masturbation-related problems	1.92	1.00	2.23	1.04	161	−1.94	> .05	.31

### Reasons for teaching sex education

No differences in reasons to teach sex education were found between paid care staff members who teach sex education and those who do not. The highest scores were given to “the client has sexuality-related questions” (*M* = 4.31, *SD* = 1.13) and “the client is exhibiting sexually inappropriate behaviour” (*M* = 4.20, *SD*) = 1.28). The lowest score was given to “the client is developmentally ready” (*M* = 3.67, *SD* = 1.13).

### Reasons for not teaching sex education

As with reasons for teaching sex education, a list of reasons for not teaching sex education was also given. Staff members who indicated that they did not teach sex education, were asked to rate to what extent the reason given would be a reason not to teach sex education, with a score of 1 being *not a reason* and 5 being *a really important reason* (see Table 4). The highest scores were given to “my clients are not sexually active” (*M* = 3.88, *SD* = 1.95), “my clients don't want to be educated” (*M* = 3.66, *SD* = 1.93), and “I don't have the knowledge and/or skills for it” (*M* = 2.58, *SD* = 1.65). The lowest scores were given to “I don't have a quiet environment to teach in” (*M* = 1.65, *SD* = 1.31) and “I don't have time for it” (*M* = 1.73, *SD* = 1.32). Additionally, the whole group was asked to respond to the statement, “teaching sex education to people with intellectual disabilities leads to more problems in the area of sexuality” (where 1 = *strongly disagree* and 5 = *strongly agree*). Both the group that teaches sex education (*M* = 1.86, *SD*) = 0.81) and the group who does not (*M* = 1.99, *SD*) = 0.75) disagreed with this statement, *t*(161) = 1.05, *p* = .30, *d* = .17.

**Table 4.** Means and standard deviations of reasons for not teaching sex education

Reasons	*M*	*SD*
I don't have time for it	1.73	1.32
I don't have a quiet environment to teach in	1.65	1.31
It's not my responsibility/task	2.13	1.78
My clients are not sexually active	3.88	1.95
I find it unpleasant to do	2.03	1.34
I am too close to my client for teaching that	1.84	1.43
I don't have the right materials	2.36	1.74
I don't have the knowledge and/or skills for it	2.58	1.65
My clients don't want to be educated	3.66	1.93

*Note. n* = 99.

### Sex education materials

An association between the presence of sex education materials and the behaviour (practice) of providing sex education was found. A chi-square test was performed on the presence of materials (yes/no) and the behaviour of providing sex education (yes/no). In the group of staff members who teach sex education, a larger portion of staff members indicated that materials are present in their working environment (78%) than in the group where staff members indicated that they do not teach sex education (58%), χ^2^(1, *N* = 163) = 7.28, *p* < .01.

Of the 64 staff members who teach sex education, 64% (*N* = 41) had used sex education materials and 36% (*N* = 23) had never used materials to teach sex education. The staff members who made use of sex education materials were on average positive about how the materials took into account the disability level of the person with intellectual disability (*M* = 3.61, *SD* = 0.86).

### Policy

In the Netherlands, the general policies of organisations that specialise in the care of people with intellectual disability are aimed towards improving the sexual health of their clients. Of all staff members who participated in this study (*N* = 163), 99% knew that policy concerning the sexuality of their clients exists within the organisation; 95% also knew where they can find this policy. When asked whether they are familiar with the content of the policy, 40% indicated that they knew nothing to a little of the content of the policy, 38% indicated that they knew most or the entire content of the policy, and 22% fell somewhere in the middle.

## Discussion

In previous studies, barriers have been identified that may affect the provision of sex education by paid care staff to people with intellectual disability (Abbott & Burns, 2007; Abbott & Howarth, 2007; Bazzo et al., 2007; Cuskelly & Bryde, 2004; Evans et al., 2009; Gilmore & Chambers, 2010; Grieve et al., 2007; Healy et al., 2009). In this study we tried to establish which factors are important factors associated with the intention of paid care staff to provide sex education and whether these results were similar to the results of previous studies.

Of the 163 paid care staff members who were included in the analyses, 39% indicated that they teach sex education. These numbers were expected to be higher, since the staff members who were included were fully responsible for the wellbeing, and therefore sexual health, of their clients, who were (young) adults with a moderate to mild intellectual disability. Furthermore, in accordance with previous studies (Abbott & Burns, 2007; Abbott & Howarth, 2007), the results imply that when sex education is provided, it is taught in response to problems, rather than to prevent problems or proactively support people with intellectual disability (Young, Gore, & McCarthy, 2012). This was reflected in the reasons for teaching sex education with the highest ratings: “the client has sexuality-related questions” and “the client exhibits sexually inappropriate behaviour.” The reason with the lowest rating was “the client is developmentally ready,” which should be the foremost reason. Also, client behaviour is an important determinant for the intention to teach sex education, meaning that the more experience staff members have with sexuality-related issues of their clients, the more inclined they are to teach sex education. This is contradictory to why most sex education programs are developed; namely, as a preventive tool to increase the chances of having a healthy sexual life (Schaafsma et al., 2013). Teaching sex education reactively can impede the individual from having pleasurable and safe sexual experiences.

The intention to teach sex education was positively associated with teaching sex education. The association of several factors with the intention to teach was tested (Fishbein & Ajzen, 2010). Self-efficacy, instrumental and experiential attitude, and injunctive and descriptive social norm were correlated with the intention to teach. The injunctive social norm produced the highest correlation with intention, a pattern that was also found in the regression analysis. No differences were found in self-efficacy and instrumental attitude between staff members who teach sex education and those who do not. These factors do not form a barrier because both groups gave high scores on both factors. This contradicts some findings from other studies where important correlates were conservative attitudes towards sexuality (Abbott & Howarth, 2007; Evans et al., 2009; Gilmore & Chambers, 2010; Meaney-Tavares & Gavidia-Payne, 2012) and lack of training concerning sexuality-related issues (Abbott & Burns, 2007; Evans et al., 2009; Grieve et al., 2007); the latter having a negative effect on self-efficacy.

Additionally, age was shown to have a small positive correlation with both instrumental and experiential attitude, meaning that older staff members seem to have a more positive attitude towards sex education. This is contrary to what has been reported in previous studies (Abbott & Burns, 2007; Abbott & Howarth, 2007; Evans et al., 2009; Gilmore & Chambers, 2010).

Furthermore, paid care staff members who teach sex education have a higher intention to do so in the near future. They also think that colleagues, managers, and parents find it more important that they teach sex education and indicate that a higher percentage of their colleagues teach sex education. They also find it easier and more pleasant to teach sex education, feel more responsible for teaching sex education, and are more informed about the protocols and policy on sexuality. Finally, they give higher scores on client behaviour, are more satisfied about the training they receive, and are more likely to have sex education materials at their disposal.

The question of causality, however, remains: Does teaching sex education cause these differences? Or do these differences make staff members more inclined to teach sex education? The injunctive social norm seems to be an important factor associated with the intention to teach sex education reactively, as attitudes toward the behaviour are very positive, and perceived skills are high. Increasing injunctive social norms should, logically, lead to an increased provision of reactive sex education. However, self-efficacy and attitudes might be different for proactive sex education. When this form of sex education is preferable for an organisation, a new needs assessment should be conducted to identify the factors associated with this specific behaviour. Additionally, one might want to investigate what proactive sex education entails and what kind of measures need to be taken to facilitate this form of sex education (Bartholomew et al., 2011).

One possible explanation for paid care staff providing sex education reactively is potentially ambivalent attitudes toward teaching sex education. Rohleder (2010) reported that educators acknowledged that people with intellectual disability should be allowed to lead fully sexual lives; however, he also described paid care staffs anxiety in being afraid that sex education might lead to potentially dangerous behaviours. Nevertheless, the participants in our study did not think so. Still, participants may have provided a socially desirable answer, not reflecting how they really felt. Another explanation for teaching sex education reactively might be that staff members just do not talk with their clients about sexuality that often (Kok, Maassen, Maaskant, & Curfs, 2009). Not talking about the subject might lead to late detection of sexual issues or sexual health needs of the client.

To identify possible barriers, staff members who did not teach sex education were given reasons for not teaching and asked to rate to what extent these applied to their situation. An interesting finding was that no environmental constraints or time constraints were a major issue for these staff members, but the reasons for not teaching sex education with the highest rates were: “my clients are not sexually active,” “my clients do not want to be sexually educated,” and “I don't have the knowledge and/or skills for it.” That clients are not sexually active should never be a reason not to teach sex education, as it still makes them vulnerable to sexual abuse. That clients do not want to be educated would indeed present a big issue; however, research should focus on the reasons why not. The last reason contradicts the high scores on self-efficacy. It could be that this reason relates to proactive sex education and the self-efficacy scores to reactive sex education.

Of the staff members who taught sex education, 78% indicated that sex education materials were present in the working environment. Only 64% of staff members actually used materials, so we assume that 36% of the sex education happens verbally. This is unexpected because we know that graphic materials are very important when communicating with people with intellectual disability. The question that needs to be asked is why don't these staff members use any graphic materials? It would be interesting to investigate these reasons in more detail. Furthermore, of the staff members who did not teach sex education, only 58% indicated that materials were present. Even though the lack of the right materials does not seem to be an important reason for not teaching sex education, not having materials in the working environment might impede the provision of sex education. Also, not having the materials might strengthen the notion that teaching sex education is not important, thus affecting the injunctive social norm.

Some studies revealed a lack of policy or clear guidelines on how to deal with the sexuality of clients within the working environment (Abbott & Howarth, 2007; Löfgren-Mårtenson, 2004), which can lead to misinterpretations of sexual behaviour, but can also instigate idiosyncratic responses to similar situations. Differences in response may lead to differences in quality of care. This study showed that although most staff members knew that there was a policy on sexuality, and knew where to find it, only 38% indicated that they had knowledge of the majority or entire content of the policy. This could suggest implementation problems of the policy within the organisation.

### Limitations

The number of participants (*N* = 630) who took part in the study was 43% and it was possible to filter out a specific group who were directly responsible for the sexual wellbeing of the client. However, the survey did not provide a description of what is meant by sex education, because this could have influenced the scores on items and consequently skewed the results. The answers are therefore totally based on what the individual staff members themselves consider constitute sex education, which can differ from one person to another. Furthermore, we do not have data from 57% of the population that received the link to the survey. Therefore we cannot rule out a potential response bias.

### Conclusion

In conclusion, if organisations want to promote sex education among people with intellectual disability, first of all that organisation needs to establish whether staff members currently teach sex education reactively or proactively. In this study we found that the injunctive social norm is an important factor associated with reactive sex education. However, the factors associated with proactive sex education can be different and therefore the approach to changing them will differ. A new needs assessment should be conducted in order to identify the relevant factors associated with that behaviour (Bartholomew et al., 2011). Second, organisations should not only look at personal factors, such as attitude, self-efficacy, and perceived social norm, but also at environmental influences, such as policy. Knowing where to find policy on sexuality is not the same as knowing what the policy entails. Implementation of policy is as important as the development of policy aimed at influencing the sexual health of people with intellectual disability in a positive way.

Finally, most programs currently lack a theory and evidence base and are not systematically developed (Schaafsma et al., 2013). In order to truly have a positive impact on the sexual lives of people with intellectual disability, it is not only necessary to have people, such as paid care staff members, to teach sex education, but it is also important to have a good quality sex education program.
